# Mast cell activation is enhanced by Tim1:Tim4 interaction but not by Tim-1 antibodies

**DOI:** 10.12688/f1000research.8132.2

**Published:** 2016-07-08

**Authors:** Binh Phong, Lawrence P. Kane

**Affiliations:** 1 Department of Immunology, University of Pittsburgh School of Medicine, Pittsburgh, USA; 2 Immunology Graduate Program, University of Pittsburgh School of Medicine, Pittsburgh, USA

**Keywords:** Tim-1, Tim-4, mast cells, phosphorylation, ribosomal S6

## Abstract

Polymorphisms in the *T cell (or transmembrane) immunoglobulin and mucin domain 1* ( *TIM-1*) gene, particularly in the mucin domain, have been associated with atopy and allergic diseases in mice and human. Genetic- and antibody-mediated studies revealed that Tim-1 functions as a positive regulator of Th2 responses, while certain antibodies to Tim-1 can exacerbate or reduce allergic lung inflammation. Tim-1 can also positively regulate the function of B cells, NKT cells, dendritic cells and mast cells. However, the precise molecular mechanisms by which Tim-1 modulates immune cell function are currently unknown. In this study, we have focused on defining Tim-1-mediated signaling pathways that enhance mast cell activation through the high affinity IgE receptor (FceRI). Using a Tim-1 mouse model lacking the mucin domain (Tim-1 ^Dmucin^), we show for the first time that the polymorphic Tim-1 mucin region is dispensable for normal mast cell activation. We further show that Tim-4 cross-linking of Tim-1 enhances select signaling pathways downstream of FceRI in mast cells, including mTOR-dependent signaling, leading to increased cytokine production but without affecting degranulation.

## Introduction

T-cell immunoglobulin and mucin domain (TIM)-1 belongs to a family of type I transmembrane proteins with essential roles in immune regulation. Tim-1 has an N-terminal IgV domain, a mucin-like domain with multiple potential sites for O-linked glycosylation, followed by a stalk domain with potential N-linked glycosylation sites, a transmembrane domain, and a short cytoplasmic tail. Tim-1 was initially identified as a cellular receptor for hepatitis A virus (HAVCR1) ^1^. The *TIM-1* gene was positionally cloned from a locus on the same chromosome but distinct from the IL-4 gene cluster that is commonly associated with Th2-biased immune responses ^2^. Polymorphisms in murine Tim-1 are associated with susceptibility to airway hyper-reactivity (AHR), a hallmark of asthma, and increased Th2 cytokine production ^2^. Similarly, polymorphisms in human TIM-1 have been linked to atopic diseases including asthma, allergic rhinitis and atopic dermatitis ^3^. The atopy connection is particularly intriguing since genetic variations in human TIM-1 modify susceptibility or resistance to allergy, but only in individuals sero-positive for HAV ^4^. These findings suggested that Tim-1 has a role in regulation of immune responses to atopic diseases.

Mechanistically, Tim-1 was shown to co-stimulate T effector cell proliferation, with preferential effects on Th2 cytokine production. Thus, the high affinity agonistic monoclonal antibody (mAb) 3B3 was reported to inhibit the induction of respiratory tolerance in an AHR model ^5^, and to enhance both T cell proliferation and cytokine production *in vitro* and *in vivo* ^5, 6^. Antibodies recognizing distinct epitopes of Tim-1 either enhanced or attenuated lung inflammation ^7^. We further showed that ectopic expression of Tim-1 in T cells stimulated *in vitro* under “neutral” conditions promoted generation of more IL-4- rather than INF-γ production ^6^. Tim-1 can also enhance NFAT/AP-1-dependent transcription in T cells activated by TCR crosslinking, suggesting that Tim-1 functions as a co-stimulatory molecule for T cell activation ^8^. Similarly, co-stimulatory function of Tim-1 was also observed after interaction of Tim-1 with its ligand Tim-4, which is primarily expressed on APCs ^9, 10^.

Regarding signaling pathways coupled to Tim-1, we showed that tyrosine 276 in the cytoplasmic tail of Tim-1 could be phosphorylated in an Lck-dependent manner. This allows for recruitment of the p85α and β subunits of the PI3K, leading to activation of the downstream kinase Akt and subsequent activation of the transcription factors NFAT and AP-1 ^8^. Administration of the agonistic Tim-1 antibody 3B3 induces expression of early activation markers CD69 and CD25 as well as IL-2 production ^8^. Other groups have demonstrated that ligation of Tim-1 by Tim-4 can activate the ERK/MAPK pathway and enhance T cell survival by up-regulating the anti-apoptotic protein BcL-x _L_^9^. Additional studies revealed that Tim-1 could co-cap with CD3 on human T cells ^11^. Tim-1 ligation on T cells has also been reported to induce tyrosine phosphorylation of the linker for activation of T cells (LAT) and the TCR-proximal Syk family tyrosine kinase Zap70 ^9^. Taken together, these findings suggested that Tim-1 may interact with proximal TCR signaling complexes.

In addition to T cells, Tim-1 also has regulatory functions on other non-immune and immune cell types. Tim-1, also known as kidney injury molecule (KIM)-1, is upregulated on renal proximal tubules and shed upon acute renal failure ^12^. Apoptotic cell recognition by Tim-1, specifically on natural killer T (NKT) cells, may induce AHR in response to respiratory syncytial virus- or ozone-induced experimental asthma ^13^. Tim-1 has recently been shown to be expressed by IL10-secreting regulatory B cells and Tim-1 signaling is required for the induction and maintenance of these cells ^14, 15^. Specifically, the Tim-1 mucin domain is required for IL-10 production in response to phosphatidylserine (PS) binding and allograft tolerance ^14, 15^. Tim-1 is also constitutively expressed on bone marrow-derived (BMMC) and peritoneal (PMC) mast cells. Cross-linking of Tim-1 by Tim-4 enhanced IgE plus antigen-stimulated (IgE/Ag) production of Th2 type cytokines ^16^. However, the mechanisms by which Tim-1 modulates mast cell functional responses are currently unknown.

Mast cells are among the first responders of immune responses against pathogens and allergens. They have the capacity to secrete a multitude of pro- and anti-inflammatory factors that regulate allergic inflammation, pathogen defense, and anti-tumor immunity ^17^. Given the genetic and functional connection of Tim-1 to allergy and hypersensitivity and the sentinel role of mast cells in atopy, it is important to determine how Tim-1 signaling contributes to the high affinity Fc receptor for IgE (FcεRI)-mediated mast cell activation. Here we demonstrate that Tim-1 promotes NF-κB and NFAT/AP1 transcriptional activation, leading to enhanced IL-6 promoter activation and cytokine production in IgE/Ag-stimulated mast cells. Using BMMCs generated from a mouse strain lacking the Tim-1 mucin domain (Tim1 ^Δmucin^), we show that this co-stimulatory effect is independent of the Tim-1 mucin domain. Finally, we show that Tim-1, in contrast with Tim-3, acts more distal to FcεRI to enhance S6 activation, without affecting proximal FcεRI signaling. Overall, our findings provide a mechanistic explanation for the co-stimulatory effects of Tim-1 signaling on FcεRI-mediated mast cell activation.

## Methods

### Antibodies and reagents

Monoclonal anti-dinitrophenyl (DNP), IgE isotype, clone SPE-7 (Cat No. D8406), DNP _32_-HSA (Cat No. A6661), anti-FLAG M2 antibody (Cat No. F1804), cyclosporine A (Cat No. C1832), and 4-Nitrophenyl N-acetyl-β-D-glucosaminide (pNAG) (Cat No. N9376) were purchased from Sigma-Aldrich (St. Louis, MO). DNP _5_-BSA (Cat No. D5050) was from Biosearch Technologies (Petaluma, CA). Monoclonal antibodies to murine Tim-1 (3B3, RMT1-10, 5G5, 5F12) and purified Tim4-Fc (the latter consists of the IgV and mucin domains of murine Tim-4 fused to the constant region of hIgG1) were obtained from Vijay Kuchroo (Harvard Medical School). Human IgG as isotype control for Tim4-Fc was purchased from Jackson ImmunoResearch Laboratories (Cat No. 009-000-003, West Grove, PA). Monoclonal antibodies to BALB/c Tim-1 (1H9, 3A2, 4A2, 4B2, 4G8, 5D1) were originally developed at Biogen and were obtained under an MTA with CoStim Pharmaceuticals (Cambridge, MA). Purified rat IgG2a as isotype control for Tim-1 antibodies was purchased from eBioscience (Cat No. 14-4321-85, San Diego, CA). Phospho-specific antibody to ERK (T202/Y204) was from BD Biosciences (20 μl per test), (Cat No. 612566, San Jose, CA). Phospho-specific antibodies to Syk (Y519/520) (1:400, Cat No. 2710), and S6 (S235/236), (1:150, Cat No. 4851) were obtained from Cell Signaling Technology (Danvers, MA). Anti-mouse Fc block (2.4G2) was purchased from BD Biosciences (Cat No. 553141, San Jose, CA). IL-6 luciferase reporter constructs were obtained from Sarah Gaffen (University of Pittsburgh), originally from Oliver Eickelberg (Helmholtz Zentrum Munchen).

### Mice

All studies were performed in accordance with University of Pittsburgh Institutional Animal Care and Use Committee procedures. Specifically, mice were housed four males or five females per polycarbonate cage in a 12-hour light/dark cycle. Food and water were provided *ad libitum*. Cages and bedding were changed every seven days. Mutant mice lacking Tim-1 mucin domain (Tim-1 ^Δmucin^) were obtained from David Rothstein (University of Pittsburgh), and were originally from Vijay Kuchroo (Harvard Medical School). Age-and sex-matched wild-type C57BL/6 were purchased from the Jackson Laboratory (Bar Harbor, ME) as control.

### BMMC and mast cell line culture

Bone marrow cells from C57BL/6 Tim-1 wild-type (WT) and mutant (Tim-1 ^Δmucin^) were generated as described previously ^18^. MC/9 mast cells were cultured in DMEM supplemented with 10% BGS, 2-ME, Pen/Strep with Glutamine, and 10% IL3-conditioned media.

### BMMC stimulation, cytokine secretion, and phospho-flow cytometry

BMMCs (1 × 10 ^5^ or 1 × 10 ^6^ cells for cytokine measurement and 3 × 10 ^5^ cells for phospho-flow) were sensitized overnight with 1 μg/ml IgE without IL-3 conditioned media. Cells were stimulated with DNP _32_-HSA or DNP _5_-BSA. Six or twenty-four hours post stimulation, supernatants were collected and assayed for murine IL-6 and TNF-α by ELISA (BioLegend). For phospho-flow staining, stimulated cells were fixed in 1.5% paraformaldehyde for ten minutes at room temperature and permeablized with ice cold methanol for thirty minutes. Incubation with phospho-specific antibodies were performed per manufacturer’s instructions. Flow acquisition was performed on Fortessa or LSRII (BD Biosciences), and data were analyzed using FlowJo software version 8.7 (Tree Star).

### Measurement of beta-hexosaminidase release

BMMCs (2.5 × 10 ^5^ cells) were sensitized and subsequently stimulated for thirty minutes in Tyrode’s buffer (135mM NaCl, 5mM KCl, 5.6mM glucose, 1.8mM CaCl _2_, 1mM MgCl _2_, 20mM HEPES, and 0.5mg/ml BSA). Supernatants (stimulated release) were collected and cells were lysed (content) with 0.5% Triton-X100 in PBS for fifteen minutes on ice. Content and stimulated release fractions were incubated with 1mM pNAG substrate for 1 hour at 37°C. Carbonate buffer (0.1M, pH 9.0) was added to stop reaction and absorbance was obtained at 405nm on a plate reader (BioTek ELx808). Percentage of beta-hexosaminidase release was calculated as (release ^stimulated^/content ^total^) × 100.

### Mast cell degranulation assay by Annexin V-based flow cytometry

Degranulation was measured as described previously ^18, 38^. Briefly, BMMCs were loaded with 0.1μM of Lysotracker Deep Red (Invitrogen) for thirty minutes at 37°C prior to sensitization with 0.5 μg/ml IgE for 1 hour. Ninety minutes after antigen cross-linking by DNP _32_-HSA, cells were collected and stained with Annexin V (BioLegend). %degranulation was determined as percentage of BMMCs that is AnnexinV ^+^Lysotracker ^lo^. Flow acquisition was performed on Fortessa or LSRII (BD Biosciences), and data were analyzed using FlowJo software version 8.7 (Tree Star).

### Transcriptional reporter luciferase assays

MC/9 mast cells (15 × 10 ^6^) were transfected with 15 μg of IL6-luc, NF-κB-luc, or NFAT/AP1-luc with 5 μg of pCDEF3 (empty vector), FLAG-tagged Tim-1 full length (FL), Tim-1 cytoplasmic deletion (Δcyto), or Tim-1 tyrosine mutant (Y276F). Electroporation was performed at 290V, 950 μF using a Gene Pulser II apparatus (Bio-Rad). Cells were collected twenty-four hours post-transfection and stimulated with 0.5 μg/ml IgE and 30 ng/ml or 100 ng/ml of DNP _32_-HSA as antigen for six hours. Luciferase assays were performed as previously described ^39^.

### Statistical analysis

All statistical analyses was performed using Prism version 6.0 (GraphPad Software). Paired, unpaired, two-tailed Student’s t-test, one-way and two-way ANOVA with multiple comparison were used for data analysis and calculation of p values, as appropriate.

## Results

**Figure 1.**
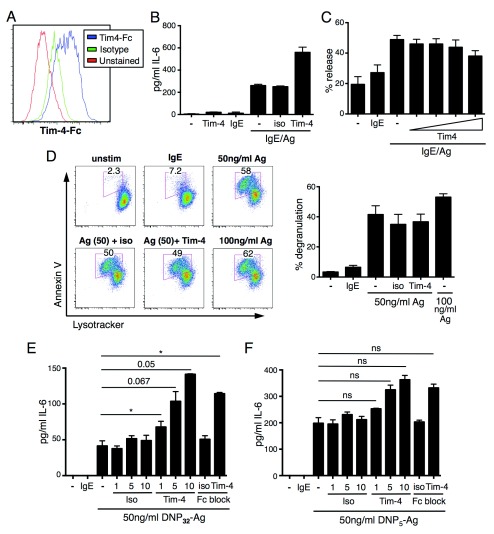
Tim4-Fc enhances IgE/Ag-mediated mast cell cytokine production at high but not low antigen valency stimulation without affecting degranulation. C57BL/6 BMMCs was incubated with 0.5 μg of Tim4-Fc or human IgG (iso) on ice for 20 minutes followed by flow cytometry analysis ( **A**). BMMCs were sensitized overnight with 1 μg/ml of IgE and stimulated with 50 ng/ml of DNP _32_-HSA, alone or with 5 μg/ml of Tim4-Fc or isotype control Fc (“iso”) for six hours prior to IL-6 measurement by ELISA ( **B**). BMMCs were sensitized with IgE and stimulated with DNP _32_-HSA in the presence of increasing amount of Tim4-Fc (0.1–50 μg/ml) for thirty minutes prior to degranulation measurement by means of beta-hexosaminidase release ( **C**). Cells were loaded with Lysotracker Deep Red, sensitized with IgE, and stimulated with antigen plus isotype control or Tim4-Fc for ninety minutes prior to Annexin V staining and flow cytometry analysis ( **D**). BMMCs were sensitized with IgE and stimulated with DNP _32_-HSA ( **E**) or DNP _5_-HSA ( **F**) alone or in conjunction with indicated amount of control Fc (“iso”) or Tim4-Fc for six hours. Anti-mouse Fc blocking Ab (2.4G2) was added as indicated for ten minutes prior to antigen and antibody stimulation. IL-6 cytokine production was determined by ELISA. Results are representative of three independent experiments with duplicates in each ( **B**, **E**, **F**), average of four experiments ( **C**), and at least three experiments ( **D**). *p<0.05.

**Figure 2.**
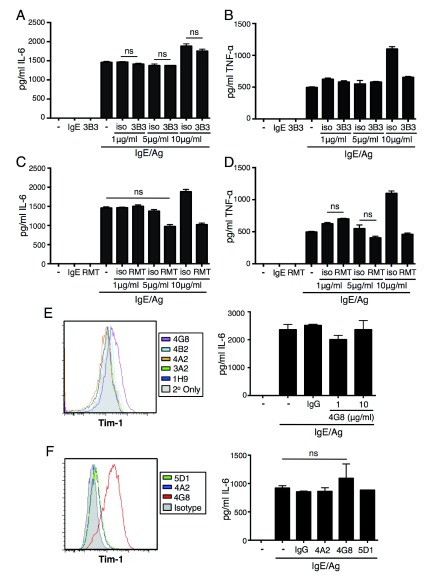
Tim-1 antibodies did not significantly alter IgE/Ag-mediated mast cell cytokine production compared to ligation by Tim4-Fc. BL/6 or BALB/c BMMCs (1 × 10 ^6^) as indicated were sensitized with IgE overnight and stimulated with DNP _32_-HSA in the presence of isotype control or monoclonal antibodies against Tim-1 3B3 ( **A**– **B**), RMT1-10 ( **C**– **D**), 3A2, 4A2, 4B2, 4G8, 1H9 ( **E**) for six hours. The indicated antibodies were incubated with BALB/c BMMCs for 30 minutes on ice followed by anti-rat IgG-Alexa-647 secondary antibody, prior to flow cytometry analysis ( **E**). Culture supernatants were analyzed for IL-6 and TNF-α by ELISA. BL/6 BMMCs were incubated with 4G8, 4A2 and 5D1 antibodies followed by anti-rat IgG-Alexa 647 secondary antibody prior to flow cytometry analysis ( **F**). BL/6 BMMCs sensitized with IgE overnight and stimulated with DNP _32_-HSA together with either isotype control or the indicated antibodies for six hours prior to IL-6 measurement by ELISA ( **F**). Results are representative of three ( **A**– **D**) and two ( **E**– **F**) independent experiments performed in duplicates.

**Figure 3.**
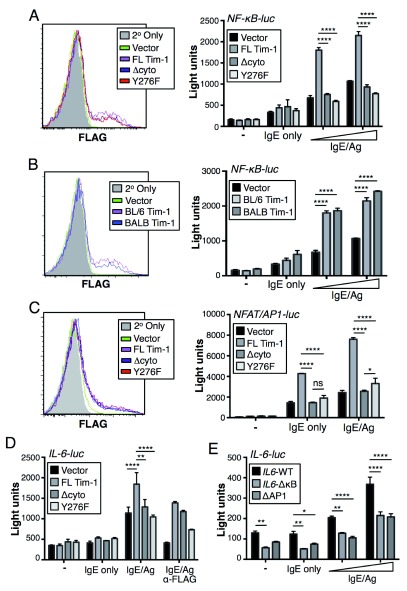
Tim1-mediated enhancement of transcriptional activation is dependent on a tyrosine on its cytoplasmic tail. MC/9 mouse mast cells were transfected with empty vector (pCDEF3), BL/6 or BALB/c Tim-1 (full length (FL)), BL/6 Tim-1 lacking the cytoplasmic region (Δcyto) or full length Tim-1 (BL/6) with tyrosine to phenylalanine mutated at tyrosine 276 (Y276). Transfected cells were stimulated with 0.5 μg/ml IgE plus either 30 ng/ml or 100 ng/ml DNP _32_-HSA, with or without addition of anti-FLAG antibody for six hours. MC/9 mast cells were co-transfected NF-κB ( **A**– **B**), NFAT/AP1 ( **C**), and IL-6 ( **D**) luciferase reporters. MC/9 mast cells were transfected with full length BL/6 Tim-1 along with indicated IL-6 luciferase reporters and stimulated as described ( **E**). Results are representative of three independent experiments performed in triplicate. *p<0.05, **p<0.005, ****p<0.00005.

**Figure 4.**
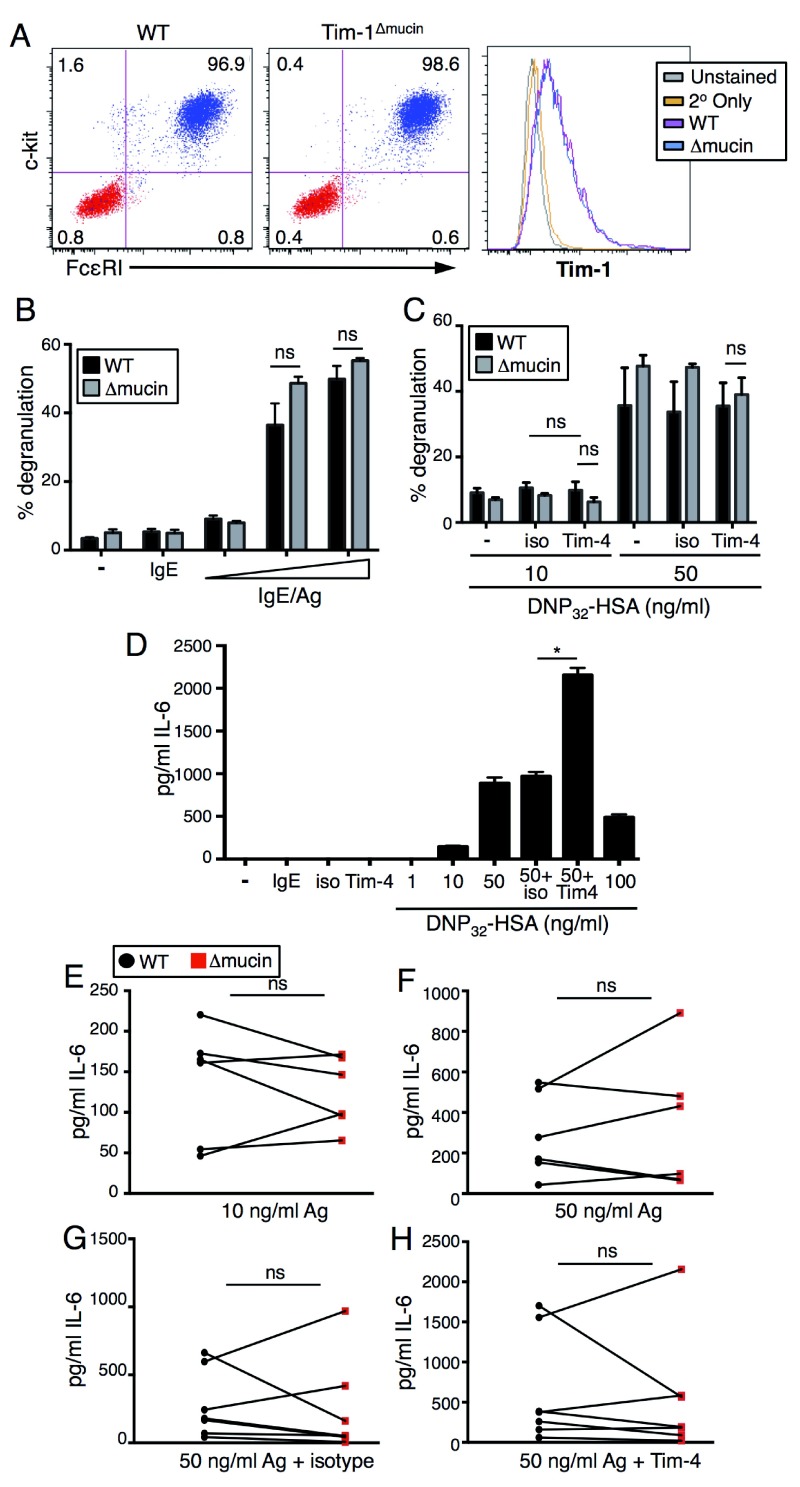
Tim-1 mucin domain is not required for Tim4-mediated enhancement of cytokine production in IgE/Ag-stimulated Tim-1 ^Δmucin^ BMMCs. Tim-1 surface expression and maturity of WT and Tim-1 ^Δmucin^ BMMCs were determined by FcεRI and c-kit staining ( **A**). WT and Tim-1 ^Δmucin^ BMMCs were sensitized overnight with IgE and stimulated with 10 ng/ml, 50 ng/ml, 100 ng/ml DNP _32_-HSA, and 5 μg/ml Tim4-Fc. Mast cell degranulation was measured by Annexin V and Lysotracker staining ( **B**– **C**). Results are average of three ( **B**) and two ( **C**) independent experiments. Culture supernatants were collected and analyzed for IL-6 by ELISA ( **D**). Using six separate batches of BMMCs. WT and Tim-1 ^Δmucin^ BMMCs were stimulated with indicated amount of antigen with either 5 μg/ml isotype control Fc (iso) or Tim4-Fc for six hours ( **E**– **H**). Results are comparison between WT and Tim-1 ^Δmucin^ BMMCs from six independent experiments using six separate batches of BMMCs. *p<0.05.

**Figure 5.**
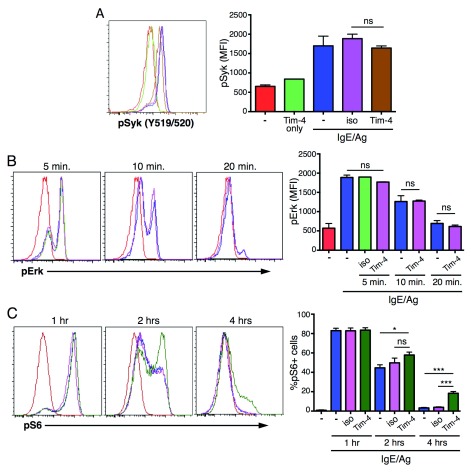
Tim-1 ligation by Tim4-Fc enhances mast cell cytokine production by sustaining ribosomal protein S6 phosphorylation upon IgE/Ag activation. BL/6 BMMCs were sensitized overnight with IgE and stimulated with DNP _32_-HSA in the presence of isotype control or Tim4-Fc for the indicated time. Syk phosphorylation (Y519/520) ( **A**), pErk (T202/Y204) ( **B**), and pS6 (S235/236) ( **C**) were analyzed by phospho-flow cytometry. Results are average of two independent experiments ( **A**– **B**) or three independent experiments ( **C**). *p<0.05, ***p<0.0005.

Raw data for Figures 1–5 in ‘Mast cell activation is enhanced by Tim1:Tim4 interaction but not by Tim-1 antibodies’ Click here for additional data file. Copyright: © 2016 Phong B and Kane LP 2016 Data associated with the article are available under the terms of
the Creative Commons Zero "No rights reserved" data waiver (CC0 1.0
Public domain dedication).

### Tim1-Tim4 interaction enhances mast cell cytokine production without affecting degranulation

Mast cells constitutively express surface Tim-1 and Tim-3, but not Tim-2 or Tim-4 ^16^. Tim-1 cross-linking by Tim4-Fc was shown to enhance cytokine production in a dose-dependent manner without affecting degranulation ^16^. We first confirmed that Tim4-Fc could bind to our cultures of bone marrow derived mast cells (BMMC) from C57BL/6 mice ( Figure 1A). We observed similar effects of Tim4-mediated IL-6 production in IgE/Ag-stimulated BMMCs ( Figure 1B). In addition, we quantified the degranulation response by measuring beta-hexosaminidase release as well as with a flow cytometry-based assay that tracks both Annexin V binding to exposed PS and loss of Lysotracker staining, due to granule release (AnnexinV+Lysotracker ^lo^). The latter method has the advantage of a high signal-to-noise ratio, and as such has been a robust assay for evaluating IgE/Ag-stimulated mast cell degranulation ^18^. Thus, we showed that varying the concentration of Tim-4, along with suboptimal antigen concentration to observe potential co-stimulation, did not have an effect on the immediate degranulation response ( Figure 1C–D). These results suggest that Tim-1 ligation by Tim-4 can exert differential effects on the immediate degranulation response, vs. late-phase cytokine production.

Antigen valency and concentration have been shown to control the outcomes of FcεRI engagement in not just quantitative, but also qualitative, fashions ^19^. Specifically, low antigen concentration or valency will activate only positive FcεRI signaling pathways, while high antigen valency or concentration will preferentially engage negative signaling components downstream of FcεRI. This is due to activity of the Src family kinase Lyn as a positive and/or negative regulator of FcεRI signaling at low or high antigen valency, respectively ^19^. We stimulated BMMCs with low (DNP _5_-BSA) or high (DNP _32_-HSA) potency antigens in the presence of Tim4-Fc or isotype control and assessed whether Tim-1 ligation could contribute to receptor signaling intensity. Thus, at high antigen valency, which also activates negative feedback of antigen receptor signaling, Tim-4 was able to maintain high IL-6 production but not at low antigen valency that induces robust antigen receptor signaling ( Figure 1E–F). Specifically, increasing the amount of Tim-4 further promoted cytokine secretion under high valency antigen stimulation, i.e. under conditions where the negative signaling loop is triggered. To exclude the possibility that Fcγ receptor binding may interfere with Tim1-Tim4 interaction, we compared IL-6 release by BMMCs, with or without addition of Fc blocking antibody, and found no differences, at least at the concentration of Tim4-Fc used throughout this study (5 μg/ml). These findings indicate that Tim-1 ligation can modulate the intensity of the antigen-induced positive FcεRI signaling pathways and may be able to bypass the negative feedback signaling loop controlled by Lyn.

### Tim-1 antibodies do not have significant effects on IgE/Ag-mediated mast cell activation

Several antibodies against Tim-1 have been tested and showed no effects on either IgE/Ag-induced mast cell degranulation or cytokine production ^16^. Of significance are the mAbs 3B3 and RMT1-10, which have been termed “agonistic” and “antagonistic” antibodies, respectively, due to their ability to enhance or inhibit effector T cell activation ^20^. Similarly, we did not observe any dose-dependent effects of 3B3, RMT1-10 or the IgV domain-binding mAb 5F12 on levels of IL-6 and TNF-α secreted by IgE-sensitized and Ag-stimulated BMMCs ( Figure 2A–D). We examined several other antibodies, generated against the BALB/c allele of Tim-1, which were shown to either exacerbate or ameliorate Th2-dependent OVA-induced lung inflammation in mice ^7^. Specifically, mAbs 1H8 and 3A2 bind to distinct epitopes near an N-linked glycosylation site in the stalk region and have agonistic and antagonistic activity, respectively. The mAb 4A2 binds to the IgV domain of Tim-1 and reduces lung inflammation and pathology ^7^. However, aside from 4G8, we did not detect significant binding of these antibodies to BALB/c Tim-1. While 4G8 recognized surface Tim-1, it did not alter Ag-mediated cytokine production in mast cells ( Figure 2E). Even though these antibodies were raised against the BALB/c allele of Tim-1, we also observed binding of 4G8 to C57BL/6 Tim-1 ( Figure 2F). Again, there was no detectable increase in IL-6 production when these antibodies were used in co-stimulation with FcεRI crosslinking by IgE/Ag, on BMMC derived from C57BL/6 mice ( Figure 2F).

### Tim-1 cytoplasmic tyrosine is required for enhancement of transcriptional activation

We previously demonstrated that transient expression of Tim-1 co-stimulated TCR/CD28-mediated transcriptional activation of IL-4 and IFN-γ production and NF-AT/AP1-dependent transcription. This co-stimulatory activity was dependent on tyrosine 276 in the Tim-1 cytoplasmic tail ^6^. Similarly, ectopic expression of an N-terminal FLAG-tagged Tim-1 on MC/9 mast cells was able to enhance IgE/Ag-stimulated NF-κB transcriptional activation ( Figure 3A). This enhancement was abrogated when the cytoplasmic tail of Tim-1 was deleted or when a tyrosine-phenylalanine mutant (Y276F) was used ( Figure 3A). Polymorphisms in Tim-1 have been associated with differential responses to OVA-induced allergic asthma ^2^. We found that both isoforms of Tim-1 (BL/6 and BALB/c) could significantly enhance activation of an NF-κB transcriptional reporter to a comparable extent ( Figure 3B). Consistent with findings in T cells and effects on mast cell cytokine production, transient expression of Tim-1 also up-regulated NF-AT/AP1 and IL-6 promoter activation, in a tyrosine phosphorylation-dependent manner ( Figure 3C–D). Furthermore, using IL-6 promoter deletion constructs, we showed that Tim-1 mediated enhancement of transcriptional activation and subsequent production of IL-6 through activation of NF-κB and AP-1 transcription factors ( Figure 3E). Unlike Tim-4 crosslinking of Tim-1, addition of anti-FLAG antibody could not further promote reporter activity, suggesting either that Tim-1 may have other ligands on MC/9 mast cells or that Tim-1 can homo-dimerize after ectopic expression, via its heavily glycosylated mucin domain, leading to downstream signaling.

### Tim-1 mucin domain is not required for Tim4-mediated enhancement of mast cell cytokine production

Our findings thus far suggest that the Tim1-Tim4 interaction augments FcεRI signaling itself, rather than acting through a parallel pathway, since Tim-4 treatment alone does not induce any detectable cytokine production or degranulation. While the Tim1-Tim4 interaction has been attributed primarily to the IgV domain, Tim-4 has also been proposed to bind to the Tim-1 mucin domain ^10^. Intriguingly, the genetic linkage of Tim-1 to allergies and asthma is associated with polymorphisms in the mucin domain. Using a mutant mouse lacking only the Tim-1 mucin domain (Tim-1 ^Δmucin^) ^21^, we determined whether the Tim-1 mucin domain is necessary to relay the co-stimulatory effects of Tim-4 binding. We noted no obvious defects in mast cell development or maturation, when BMMC were generated from Tim-1 ^Δmucin^ bone marrow. Thus, WT and Tim-1 ^Δmucin^ BMMCs expressed comparable levels of surface Tim-1, based on staining with IgV-binding Tim-1 antibodies ( Figure 4A). Tim-1 ^Δmucin^ BMMCs exhibited a similar extent of degranulation in response to antigen stimulation, compared to WT BMMCs, which was not altered by Tim-1 ligation ( Figure 4B–C). Next, we examined the ability of Tim-1 ^Δmucin^ BMMCs to secrete cytokines in response to IgE/Ag. Tim-1 ^Δmucin^ mast cells appeared to respond normally to antigen stimulation, and also to Tim4-mediated co-stimulation ( Figure 4D). Using different batches of BMMCs over the course of multiple experiments, we were not able to consistently observe any major difference between WT and Tim-1 ^Δmucin^ BMMCs. We did observe some variation in the amount of IL-6 produced by different batches of BMMCs, but this appeared to be largely due to the relative maturation status of the cells ( Figure 4E–H). These results indicated that the mucin domain of Tim-1 is not required for normal mast cell responses and also that Tim-4-mediated mast cell activation does not appear to involve the mucin domain of Tim-1, but rather its IgV domain.

### Tim-1 enhances Ag-activated phosphorylation of ribosomal protein S6

Next, we assessed the potential signaling pathways utilized by Tim1-Tim4 interaction downstream of FcεRI signaling to upregulate mast cell cytokine production. Using a Nur77GFP reporter mouse, we previously showed that unlike the related family member Tim-3, engagement of Tim-1 did not enhance FcεRI signal intensity, thereby showing that Tim-1 cross-linking does not augment antigen receptor-proximal signaling ^18^. We moved on to explore signaling pathways both proximal and distal to the FcεRI complex, for any effects of Tim-1. Thus, the Zap70-related kinase Syk is an FcεRIγ-associated activator integral to activation of LAT, SLP76, PLC-γ and other adaptor molecules essential for signal transduction downstream of FcεRI ^22^. Phosphorylation of Syk was not further increased by Tim-4 ( Figure 5A). Similarly, we observed robust phosphorylation of ERK and Akt upon IgE/Ag-induced activation that was not affected by Tim-1 engagement ( Figure 5B and data not shown). These results demonstrate that MEK/ERK and PI3K/Akt pathways are not involved in enhancement of Ag-mediated mast cell function by Tim-1, even though we observed that phosphorylation of Tim-1 cytoplasmic tail by TCR activation led to recruitment of p85 subunit of PI3K ^8^. Nevertheless, we did detect a significant increase in phosphorylation of ribosomal protein S6, an important target of the PI3K/mTOR pathway regulating cell growth, survival, metabolism, and protein synthesis in mast cells ^23^. Mast cells exhibited robust phosphorylation of S6 (~80% of BMMCs) one hour post-Ag stimulation, which did not increase further with Tim-4 addition. However, Tim-4 treatment was able to maintain a significant percentage of pS6-positive BMMCs for as long as four hours, even as the Ag-triggered signal returned to basal levels ( Figure 5C). Overall, these results support a positive role of Tim-1 activation by Tim-4 to sustain mTOR-dependent mast cell metabolism and protein synthesis, leading to enhanced cytokine production.

## Discussion

Mast cells constitutively express high levels of cell-surface Tim-1, a molecule with co-stimulatory effects on many immune cell types, but with unclear mechanisms of action. Here we demonstrate that Tim-1 is a positive regulator of mast cell activation and cytokine production. Similar to our findings on the effects of Tim-3 on mast cells ^18^, ectopic expression of Tim-1 expression was sufficient to promote IgE/Ag-mediated NF-κB and NF-AT/AP1 transcriptional activation (at least in MC/9 cells), without additional cross-linking antibodies or exogenous ligands. Tim-4 is a ligand for Tim-1, but the lack of Tim-4 expression on mast cells makes it an unlikely explanation for this particular role of Tim1 in mast cells. Tim-1 can also bind PS on apoptotic cells ^24^ or PS transiently exposed on degranulating mast cells, either of which could potentially contribute to enhance Tim-1 signaling, although whether PS binding to Tim-1 can lead to mast cell activation is still unknown. Tim-1 has also been reported to bind LMIR5/CD300b, an activating receptor expressed on myeloid cells ^25^. Stimulation with Tim1-Fc was able to induce LMIR5-mediated ERK activation in mast cells, suggesting that LMIR5 is an endogenous ligand of Tim-1, driving the enhancement of transcriptional response. Finally, Tim-1 may homodimerize through its glycosylated mucin-like domain, leading to downstream signaling and function. We showed that Tim-1 co-stimulation is dependent on the tyrosine phosphorylation motif in its cytoplasmic tail, as mutation of tyrosine 276 rendered Tim-1 unable to mediate co-stimulation. The Src kinase Fyn has been shown to phosphorylate Tim-1 in a B cell line ^26^. We showed that the Tim-1 cytoplasmic tail is phosphorylated upon TCR stimulation in an Lck-dependent manner and can recruit p85 binding ^8^. Therefore, Src family kinases like Lyn, Fyn or Hck are potential facilitators of Tim-1 phosphorylation upon IgE/Ag activation in mast cells.

Another aim of this study was to determine how Tim-1 cross-linking by antibodies or ligands could modulate mast cell activation. Unlike in T cells or *in vivo* experiments, mast cells did not respond to Tim-1 antibody treatment, as none of the antibodies tested elicited a change in mast cell degranulation or cytokine production. On the other hand, Tim-4 treatment consistently enhanced IgE/Ag-mediated cytokine production but not degranulation, which may be a time- and/or signal intensity-dependent effect. Tim-4 was reported to have bimodal effects, either enhancing or inhibiting T cell proliferation, depending on anti-CD3/CD28 concentrations ^10^. This bimodal regulation was later reported to inhibit activation of naïve T cells, which do not express Tim-1, and to enhance activation of effector T cells, suggesting that Tim-4 binds to an unknown ligand expressed preferentially on naïve T cells ^27^. In addition, Tim-4 can bind to naïve T cells that do not express Tim-1 and inhibit Th17 differentiation ^28^. This effect was shown to be independent of Tim-1 activity, since addition of Tim-1 blocking antibody, presumably to block the Tim1:Tim4 interaction, could not rescue Tim4-mediated inhibition ^28^. In contrast to such ligand-dependent effects observed on T cells, Tim-4 co-stimulates IgE/Ag-mediated mast cell activation by cross-linking Tim-1. Using low and high valency antigens to engage the positive and negative signaling pathways of FcεRI, respectively, we showed that Tim-4 could enhance mast cell cytokine production in both settings. Specifically, Tim-4 co-stimulated cytokine release in a dose-dependent manner, under both high and low valency antigen stimulation. These results imply that Tim-4 contributes to FcεRI signaling intensity and/or duration, and may potentially override negative feedback signals linked primarily to Lyn-mediated phosphatase activation. Together with findings that Tim-4 alone does not induce cytokine production in mast cells, our results demonstrate that Tim-1 signaling interfaces with common effector molecules downstream of FcεRI signaling, rather than acting through a parallel pathway, to enhance mast cell functions.

To determine whether Tim-1 plays a positive or negative regulatory role in mast cells, we first attempted siRNA-mediated knockdown of Tim-1 protein in BMMCs but were unsuccessful in obtaining efficient reduction of Tim-1 expression. Furthermore, two separate strains of Tim-1-deficient mice showed relatively unaltered IgE production and AHR development in an OVA-induced mouse model of asthma, although one study did note higher type 2 and Th17 cytokine production in Tim-1 knockout (KO) mice ^26, 29^. On the other hand, the importance of the Tim-1 mucin domain has been demonstrated in T cell activation, differentiation, trafficking, and effector function in autoimmunity and airway inflammation ^28, 30^. The mucin domain of Tim-1 is also essential for homeostasis and function of regulatory B cells ^14, 15, 21^. Thus, we examined whether the Tim-1 mucin domain regulates mast cell activity, particularly in the context of Tim-4 treatment. Contrary to the effects seen in B and T cells, the mucin domain was dispensable for the effects of Tim-1 in mast cells, as degranulation and cytokine release were intact in the absence of the mucin domain. It is worth noting that Tim-1 ^Δmucin^ BMMCs were actually able to secrete more cytokine than WT BMMCs in some instances. However, this was not a consistent finding. While our study focused on bone marrow-derived mast cells, absence of the Tim-1 mucin domain may nonetheless affect the differentiation and/or function of other mast cell types in their respective tissue microenvironments *in vivo*. Tim1-Tim4 interactions are thought to occur mostly through their respective IgV domains, although there is evidence that Tim-4 may also bind to the Tim-1 mucin domain ^10^. We showed that Tim-4 mediated co-stimulation of mast cell function occurred independent of the Tim-1 mucin domain. It remains to be determined, in the absence of the Tim-1 mucin domain, whether Tim-4 has other unknown ligands on mast cells that can mediate this enhancement.

We also examined the signaling pathways upstream of enhanced transcriptional activation and cytokine production by Tim-1 and Ag co-stimulation in mast cells. Phosphorylation of Syk was not altered by Tim-4 treatment at the time points we examined. This is consistent with our finding that the Tim1-Tim4 interaction did not alter Ag-stimulated FcεRI signaling intensity, using mast cells from a Nur77 ^GFP^ mouse model ^18^. Syk is phosphorylated on multiple tyrosines by either auto-phosphorylation or trans-phosphorylation by Lyn, resulting in enhancement or inhibition, respectively, of Ag-mediated FcεRI signaling ^31– 33^. Since we only examined tyrosines 519 and 520 in the Syk activation loop, which are sites of Syk auto-phosphorylation, it is possible that other tyrosine phosphorylation sites of phosphorylation may be affected by Tim-1. One particular site of interest is tyrosine 346 in the linker region, which is important for PLC-γ1 binding and phosphorylation ^34^. Aside from positive signaling pathways, the relevant negative signaling pathways should be investigated for potential down-regulation by Tim-1 engagement. In particular, Src homology-2-containing signaling protein (SHIP), which has been implicated in regulation of IgE/Ag-induced IL-6 production through inhibition of NF-κB activity, both of which are enhanced by Tim-1 cross-linking in our study ^35^.

In a previous study, Tim-4 was shown to enhance phosphorylation of Erk and Akt in CD4 T cells ^9^. These effects were not observed in a previous study on mast cells, with either BMMC’s or peritoneal mast cells (PMCs) ^16^. Similarly, we did not observe effects of soluble Tim-4 on these signaling pathways, in conjunction with Ag-mediated FcεRI aggregation. It is possible that Tim-4 signals are cell-type specific or that mast cells require more robust aggregation of Tim-1 at the time of antigen stimulation to induce substantial effects. While phosphorylation of Akt was not detectably affected, phosphorylation of ribosomal protein S6 was significantly enhanced upon Tim-4 treatment, an effect that correlated with enhanced cytokine production. Ribosomal protein S6 is a downstream effector of PI3K/mTOR signaling, and as such is essential for mast cell survival, proliferation, metabolism and protein synthesis. MALT1 and BCL10, members of the Carma1-MALT1-Bcl10 (CBM) complex, are essential regulators of FcεRI-induced mast cell activation by selectively up-regulating NF-κB-dependent cytokine production without affecting degranulation and leukotriene synthesis ^36^. We previously identified a Carma1-MALT1-dependent activation of mTOR signaling after TCR engagement, leading to phosphorylation of S6 and another mTOR substrate 4E-BP1 ^37^. Thus, Tim-1 may preferentially engage the MALT1-Bcl10 pathway to modulate mTOR signaling and NF-κB responses without affecting FcεRI-proximal signaling. Consequently, further studies are needed to address whether Tim1-Tim4 interaction promotes mast cell metabolic responses and protein synthesis as well as whether the specific Tim-1 targets in this pathway leading to effector functions.

## Data availability

The data referenced by this article are under copyright with the following copyright statement: Copyright: © 2016 Phong B and Kane LP

Data associated with the article are available under the terms of the Creative Commons Zero "No rights reserved" data waiver (CC0 1.0 Public domain dedication). 

*F1000Research*: Dataset 1. Raw data for Figure 1– Figure 5 in ‘Mast cell activation is enhanced by Tim1:Tim4 interaction but not by Tim-1 antibodies’, 10.5256/f1000research.8132.d114870 ^40^
