# Supplementary material for: Mast cell activation is enhanced by Tim1:Tim4 interaction but not by Tim-1 antibodies
Source: F1000Res. 2016 Jul 8;5:251. Originally published 2016 Mar 1. [Version 2] doi: 10.12688/f1000research.8132.2 (PMC5584026; doi:10.12688/f1000research.8132.2)
Supplement: Peer review history

# Peer review: Referee response for version 2

**DOI:** 10.5256/f1000research.9877.r14911

**Published:** 2016-07-11

**Authors:** Bridget S Wilson

**Affiliations:** 1 Department of Pathology, University of New Mexico, Albuquerque, NM, Mexico

I have read this submission. I believe that I have an appropriate level of expertise to confirm that it is of an acceptable scientific standard.

# Peer review: Referee response for version 2

**DOI:** 10.5256/f1000research.9877.r14838

**Published:** 2016-07-11

**Authors:** Avery August

**Affiliations:** 1 Department of Microbiology and Immunology, Cornell University, Ithaca, NY, USA

The authors have satisfied my concerns.

I have read this submission. I believe that I have an appropriate level of expertise to confirm that it is of an acceptable scientific standard.

# Peer review: Referee response for version 1

**DOI:** 10.5256/f1000research.8748.r14555

**Published:** 2016-06-23

**Authors:** Bridget S Wilson

**Affiliations:** 1 Department of Pathology, University of New Mexico, Albuquerque, NM, Mexico

The TIM-1 protein has been implicated in regulation of T cells, NKT cells and regulatory B cells.   Here, the focus is on mast cells, where Tim-1 has been shown to be constitutively expressed and is believed to have a co-stimulatory function for NF-kB and NFAT/AP1 transcription and cytokine production. 

 A few points would be helpful to address in the final version of the manuscript.

1. Prior work has shown that Tim-1 recognizes phosphatidylserine on apoptotic cells and that the mucin domain is important for this binding. Since the data presented here show that annexin-binding (which detects PS) is upregulated as a function of mast cell degranulation, the Tim-1 mucin domain might mediate homotypic interactions between recently degranulated cells.  Is there evidence that this occurs (microscopy images or flow-based measures?) If this is a mechanism to cluster Tim1 for activation, it will be in a “synapse”-like setting and would support a post-degranulation, delayed co-stimulatory signal. As mentioned below, Tim-4 mediated activation *in vivo* is probably in a synapse setting too, so there seems like a missed opportunity here.
2. This study relies a lot on exogenous agents (Tim4-Fc, antibodies) to make conclusions about the role of Tim-1 signaling. This is fine as an experimental approach, but can confound the conclusions reached about the role of the mucin domain. In other words, if one bypasses the mucin domain by activating with Tim4-Fc, it does not necessarily mean that ligations mediated via the mucin domain does not mediate signaling in another setting. I find, in general, that the emphasis on this result is overstated/over-discussed as a physiological relevant finding. It provides more insight on the structural features of Tim-1, which can apparently be activated by crosslinking (ie antibodies) or  by binding to another Tim (Tim4) presented on another cell… and possibly when PS, presented on another mast cell or an apoptotic cell in the inflammatory microenvironment, clusters Tim-1 via the mucin domain.
  In keeping with this, I think the discussion could be shortened and simplified.  For example, this text could be omitted or reworded:
  “Thus, we examined whether the Tim-1 mucin domain regulates mast cell activity, particularly in the context of Tim-4 treatment. Contrary to the effects seen in B and T cells, the Tim-1 mucin domain is dispensable for mast cell activity, as mast cell degranulation and cytokine release remain intact in the absence of the mucin domain. It is worth noting that Tim-1 [] mucin BMMCs were actually able to secrete more cytokine than WT BMMCs in some instances. However, after testing six different batches of WT and Tim-1mucin BMMCs, it appeared that any differences observed were due to the maturation status of BMMCs and their Fc[epsilon]RI surface expression, rather than any direct effect of deletion of the Tim-1 mucin domain.”
3. This group has opted to use SPE-7 (anti-DNP) IgE to prime the BMMC. Since this is a cytokinergic IgE, it could be good to confirm that the essential findings of co-stimulation also occur when using  a non-cytokinergic (anti-DNP) IgE …perhaps the Liu IgE.

I have read this submission. I believe that I have an appropriate level of expertise to confirm that it is of an acceptable scientific standard.

# Response

**Published:** 2016-06-30

**Authors:** Larry Kane

**Affiliations:** Department of Immunology, University of Pittsburgh, USA

1. *Prior work has shown that Tim-1 recognizes phosphatidylserine on apoptotic cells and that the mucin domain is important for this binding. Since the data presented here show that annexin-binding (which detects PS) is upregulated as a function of mast cell degranulation, the Tim-1 mucin domain might mediate homotypic interactions between recently degranulated cells.  Is there evidence that this occurs (microscopy images or flow-based measures?) If this is a mechanism to cluster Tim1 for activation, it will be in a “synapse”-like setting and would support a post-degranulation, delayed co-stimulatory signal. As mentioned below, Tim-4 mediated activation is probably in a synapse setting too, so there seems like a missed opportunity here.*
  - The reviewer raises an interesting point regarding the regulation of Tim-1 by PS on degranulating mast cells (although to our knowledge, the Tim1:PS interaction mainly occurs through the Tim-3 IgV domain). We have not yet been able to image Tim-1 during mast cell activation, but feel that this would be an interesting topic for future exploration.
2. *This study relies a lot on exogenous agents (Tim4-Fc, antibodies) to make conclusions about the role of Tim-1 signaling. This is fine as an experimental approach, but can confound the conclusions reached about the role of the mucin domain. In other words, if one bypasses the mucin domain by activating with Tim4-Fc, it does not necessarily mean that ligations mediated via the mucin domain does not mediate signaling in another setting. I find, in general, that the emphasis on this result is overstated/over-discussed as a physiological relevant finding. It provides more insight on the structural features of Tim-1, which can apparently be activated by crosslinking (ie antibodies) or  by binding to another Tim (Tim4) presented on another cell… and possibly when PS, presented on another mast cell or an apoptotic cell in the inflammatory microenvironment, clusters Tim-1 via the mucin domain.*
  - This is a fair point.We have modified the discussion of these results in line with this comment and the one below.
3. *In keeping with this, I think the discussion could be shortened and simplified.  For example, this text could be omitted or reworded:*
* “Thus, we examined whether the Tim-1 mucin domain regulates mast cell activity, particularly in the context of Tim-4 treatment. Contrary to the effects seen in B and T cells, the Tim-1 mucin domain is dispensable for mast cell activity, as mast cell degranulation and cytokine release remain intact in the absence of the mucin domain. It is worth noting that Tim-1 d.mucin BMMCs were actually able to secrete more cytokine than WT BMMCs in some instances. However, after testing six different batches of WT and Tim-1* d. *mucin BMMCs, it appeared that any differences observed were due to the maturation status of BMMCs and their Fc[epsilon]RI surface expression, rather than any direct effect of deletion of the Tim-1 mucin domain.”*
  - This is a good suggestion – we have now edited the Discussion, to make it more readable, and as a result it has been shortened by about one page.
4. *This group has opted to use SPE-7 (anti-DNP) IgE to prime the BMMC.  Since this is a cytokinergic IgE, it could be good to confirm that the essential findings of co-stimulation also occur when using  a non-cytokinergic (anti-DNP) IgE …perhaps the Liu IgE.*
  - We have only used the commercially available, and widely employed,SPE-7 IgE in our studies. We agree that branching out to other IgE’s would be an interesting avenue for future follow-up exploration.

# Peer review: Referee response for version 1

**DOI:** 10.5256/f1000research.8748.r12697

**Published:** 2016-03-15

**Authors:** Avery August

**Affiliations:** 1 Department of Microbiology and Immunology, Cornell University, Ithaca, NY, USA

There are a few issues that the authors should address as they refine the presentation of this work as detailed below:

1. The authors should refer to the data in Figure 1A, the characterization of the TIM-4-Fc reagent and expression of TIM-1 on the surface of these mast cells.
2. Since IgE alone has been shown to affect mast cell survival, does TIM-4/TIM-1 affect the mast cell response to IgE alone?
3. In figure 1E, the graph looks as if there is production of IL-6 in the absence of IgE (third bar from the left). Is this correct?
4. The location of the flag tag on TIM-1 should be described by the authors.
5. Do the antibodies against TIM-1 affect the ability of TIM-4 to enhance cytokine production?
6. The reference to a small although statistically insignificant increase in IL6 when 4G8 was used is not appropriate to say, since the change is not significant, and not any larger than other changes that are not remarked upon by the authors. This statement should be modified.
7. In experiments where TIM-1 is overexpressed in the MC/9 cell line, what is the ligand for the transfected TIM-1?
8. Is calcium signaling affected by TIM-4/Tim-1 interaction? Is activation of Btk affected given the potential role of PI3K downstream of TIM-1?
9. The statement in the 6 ^th^ line of the discussion starting with “Tim-1 expression alone could promote IgE/Ag-mediated NF- **k**B and NF-AT/AP1 transcriptional activation…” should be qualified that this is in MC/9 cells, which may not behave like primary mast cells.

I have read this submission. I believe that I have an appropriate level of expertise to confirm that it is of an acceptable scientific standard, however I have significant reservations, as outlined above.

# Response

**Published:** 2016-06-30

**Authors:** Larry Kane

**Affiliations:** Department of Immunology, University of Pittsburgh, USA

1. *The authors should refer to the data in Figure 1A, the characterization of the TIM-4-Fc reagent and expression of TIM-1 on the surface of these mast cells.*
  - This is now added to the Results, with an extra description of the Tim4-Fc in the methods
2. *Since IgE alone has been shown to affect mast cell survival, does TIM-4/TIM-1 affect the mast cell response to IgE alone?*
  - We did not test the IgE+Tim4-Fc condition ourselves but a previous paper from Galli and colleagues (ref. 16 in the manuscript) showed that IgE + rmTim-4 did not trigger degranulation, cytokine production, or prevent mast cell apoptosis from IL-3 withdrawal.
3. *In figure 1E, the graph looks as if there is production of IL-6 in the absence of IgE (third bar from the left). Is this correct?*
  - That condition actually contains both IgE and the indicated antigen; the bar to the left of it represents cells receiving only IgE and nothing else.
4. *The location of the flag tag on TIM-1 should be described by the authors.*
  - The tag is at the N-terminus. This is now stated explicitly in the Results section.
5. *Do the antibodies against TIM-1 affect the ability of TIM-4 to enhance cytokine production?*
  - We did not perform any blocking experiments with the Tim-1 antibodies in the presence of Tim-4 fusion protein, but this would be an interesting future experiment.
6. *The reference to a small although statistically insignificant increase in IL6 when 4G8 was used is not appropriate to say, since the change is not significant, and not any larger than other changes that are not remarked upon by the authors. This statement should be modified.*
  - This is a good point. We have now modified the sentence in question which refers to Fig. 2F in the results section to make it more accurately and clearly reflect the data.
7. *In experiments where TIM-1 is overexpressed in the MC/9 cell line, what is the ligand for the transfected TIM-1?*
  - In our previous publications, we observed what appears to be constitutive activity of the transfected Tim-1 after transient transfection. We have generally interpreted this to indicate that ectopically expressed Tim-1 is oligomerizing in the absence of ligand, since it is expressed at higher than normal levels on at least a subset of the transfected cells.
8. *Is calcium signaling affected by TIM-4/Tim-1 interaction? Is activation of Btk affected given the potential role of PI3K downstream of TIM-1?*
  - Given the data we have presented in the manuscript, it is indeed logical that a Tec family kinase like Btk may be a proximal target for Tim-1. We have started to address this by probing the effects of Tim-1 on PLC-g1 phosphorylation, but have not been able to observe a robust effect. However, given the limited personnel available to work currently on this project, we thought that it was important to share the results that we have obtained, which may be of interest to others studying the function of Tim-1.
9. *The statement in the 6 ^th^ line of the discussion starting with “Tim-1 expression alone could promote IgE/Ag-mediated NF- **k**B and NF-AT/AP1 transcriptional activation…” should be qualified that this is in MC/9 cells, which may not behave like primary mast cells.*
  - This is a good point. We have now clarified in the Discussion that this was in MC/9 cells.
